# Randomized Clinical Trial of a Topical Botanical Patch for the Adjunctive Management of Periodontitis

**DOI:** 10.3290/j.ohpd.b3147141

**Published:** 2022-06-20

**Authors:** Rebecca Wilder, William Levine, David W. Paquette

**Affiliations:** a Professor, Associate Dean for Professional Development and Faculty Affairs, Adams School of Dentistry, University of North Carolina, Chapel Hill, NC, USA. Trial management including patient recruitment, data collection, data interpretation, proofread the manuscript.; b Chief Scientific Officer, Izun Pharmaceuticals, Jerusalem, Israel. Idea, hypothesis, experimental design, data interpretation, proofread the manuscript.; c Professor and Chair, Department of Surgical Sciences, Interim Associate Dean of Research, School of Dental Medicine, East Carolina University, Greenville, NC, USA. Idea, hypothesis, experimental design, data interpretation, wrote the manuscript.

**Keywords:** botanical, clinical trial, inflammation, periodontal disease, periodontitis

## Abstract

**Purpose:**

This randomized, controlled clinical trial aimed to evaluate the clinical, adjunctive effects of an approved botanical barrier device or patch on probing parameters in patients with periodontitis.

**Materials and Methods:**

Eighty patients with periodontitis were recruited for this single-blinded trial. Patient demographic data, including gender, age, self-reported smoking status, and history of diabetes or cardiovascular disease, were collected. At baseline, all patients received a full-mouth probing examination followed by scaling and root planing (SRP). Thereafter, patients were randomized to receive either adjunctive botanical patch applications (i.e. at 2–4 treatment sites with baseline pocket depth PD ≥6 mm) or no additional therapy (SRP alone, control). Patients applied botanical patch devices per randomization to treatment sites three times on day 0 and once daily on days 1–6. Study devices were spontaneously shed or removed by the patient at 2–2.5 h after each application. Patients were recalled for probing reexaminations at 1, 2 and 3 months. Statistical analyses focused on intergroup differences in probing parameters and included ANOVA for baseline measures and ANCOVA controlling for baseline measures at 1, 2 and 3 months in the overall population and in subpopulations (e.g. smokers vs nonsmokers).

**Results:**

Randomized patient groups were balanced with respect to baseline periodontal status (mean and extent PD) but not smoking, with statistically significantly more smokers clustering in the control group (p = 0.002). For the overall population and the non-smoking subpopulation, statistically significantly improved PD and clinical attachment levels (CAL) were observed with adjunctive botanical patch therapy vs control at 1 and 2 months (p < 0.05) but not 3 months (p = 0.08 for PD). For smokers, no statistically significant intergroup differences in PD or CAL were detected with botanical patch treatment.

**Conclusions:**

The data from this trial indicate short-term improvements in probing parameters with the botanical patch device when used adjunctively with SRP, especially with non-smoking periodontitis patients.

Periodontitis is a common condition in human populations and is initiated by a dysbiosis of the oral microbiome, leading to inflammatory events and the destruction of tooth supporting tissues (alveolar bone, periodontal ligament and connective tissues).^5,11,20,24^ Data from population cohort studies emphasize the importance of controlling inflammation in the long-term management of periodontitis, prevention of disease progression, and retention of teeth.^1,12,16^ In addition, clinical trials consistently indicate that pharmacologic agents targeted at suppressing inflammatory pathways when used adjunctively with scaling and root planing (SRP) can improve surrogate outcomes related to periodontitis – such as alveolar bone loss, clinical attachment level (CAL) or pocket depth (PD) – greater than SRP alone.^2,17,25^ A recent systematic review that included 58 randomized, placebo-controlled clinical studies affirmed that local administration of 1.2% statin gels as adjuncts to nonsurgical periodontal therapy significantly improved PD reduction in infrabony defects, and systemic administration of sub-antimicrobial dose doxycycline (SDD) in addition to nonsurgical SRP improved PD reduction of deep pockets.^6^

A topical, botanical ‘patch’ or barrier device (PerioPatch, Izun Pharmaceuticals; Jerusalem, Israel) has regulatory approval from the US Food and Drug Administration (FDA) and European Union (EU) for the management of oral wounds, injuries, and ulcerations involving gingiva or oral mucosa in patients. Botanical patch devices are elliptical in shape (24 mm x 8 mm x 190 μm) and packaged in a six-unit prescription-dose pack. Following application of botanical patch devices to gingival or mucosal tissues, the protective ethyl cellulose backing is shed within 2 h, but an adhesive hydrogel film may remain in place for up to 5 h. While patch devices contain a blend of extracts from three plants (*Centella asiatica*, *Echinacea purpurea* and *Sambucus nigra*) that have anti-inflammatory properties,^9,14,18^ the devices appear to foster repair and wound healing by absorbing the local inflammatory exudate from the inflamed tissue and protecting tissues from further irritation.^3^

Topical botanical patch devices have been studied in a variety of oral conditions in human patients. Grbic et al^8^ tested the site-specific effects of botanical patch devices in patients with plaque-induced gingivitis. Fifty-three medically healthy adults with mean gingival index (GI) scores^13^ of 1.0 or greater (maxillary posterior teeth) were recruited for this proof-of-concept trial. Following the collection of baseline indices and crevicular fluid, patients were randomized for botanical patch vs placebo device applications over three days. Accordingly, botanical patch applications significantly decreased mean GI scores in patients at days 4 and 15 as compared to placebo devices. In addition, significantly more sites responded with reduced GI scores with botanical patch devices vs the placebo over the 15-day period. When the investigators measured concentrations of the inflammatory biomarker, β-glucuronidase, in crevicular fluid, they noted significantly lower levels for botanical patch vs placebo patients at days 4 and 8. Two subsequently published case series document effective clinical results when botanical patch devices were used in patients with intraoral traumatic wounds, periodontitis or peri-implant mucositis.^15,19^ In one series involving nine patients with moderate to severe chronic periodontitis, treatment with a combination of SRP plus botanical patch devices consistently reduced PD and bleeding scores (mean reductions of 2.8 mm and 94%, respectively) over 4-6 weeks.^15^ Although the available published studies to date are limited in duration and design, they lend credence to the hypothesis that the botanical patch devices reduce local inflammatory signs.

The aim of the present study was to evaluate the clinical benefits of adjunctive botanical patch therapy in patients with periodontitis as assessed with conventional probing parameters. The primary hypothesis was that the combination of SRP plus botanical patch devices would improve PD beyond SRP alone in patients with periodontitis. The secondary hypothesis was that adjunctive botanical patch therapy would improve CAL beyond SRP alone.

## Materials and Methods

### Study Design and Sample Size Estimation

The study was designed as a single (examiner) blinded, two-arm, parallel design, randomized, controlled clinical trial. The study was approved by the Biomedical Institutional Review Board at the University of North Carolina at Chapel Hill (UNC-CH) and registered with ClinicalTrials.gov.

For this study, sample size was estimated using the formula for normally distributed means.^4^ With an alpha set at 0.05 (two-sided), power at 80%, and accounting for a 10% drop-out rate, 40 patients per group were planned to detect a difference of 0.6 mm in PD and with a standard deviation of 0.9.^26^

### Patient Inclusion and Exclusion Criteria

Eighty patients, at least 18 years of age and with periodontitis, were recruited from the population of patients presenting for dental care at UNC-CH Adams School of Dentistry. For inclusion, dentate patients (i.e. with ≥12 teeth) had to be medically healthy or stable, present with at least two periodontal pockets measuring 6 mm or more on separate teeth and with bleeding on probing (BOP) at baseline. Patients meeting these probing inclusion criteria presented with stage III or IV periodontitis (any grade) according to the 2017 American Academy of Periodontology (AAP) and European Federation of Periodontology (EFP) classification of periodontal diseases and condions.^23^ Patients taking anti-inflammatory drugs, antibiotics (within 3 months of screening), drugs known to affect periodontal status (e.g. phenytoin, calcium antagonists, cyclosporine) or botanical supplements were excluded. In addition, patients with allergies to botanical products were excluded.

### Study Outcome Measures

Patient demographic data included gender, race, ethnicity, and self-reported history of diabetes or cardiovascular disease. Smokers self-identified as those using tobacco products within the last six months.^26^ Body mass index (BMI) was measured and calculated at baseline as weight (kg) divided by height (m) squared.

Prior to any study procedures, clinical examiners were trained on measuring all trial outcomes and calibrated to achieve > 90% intra- and inter-examiner agreement for the primary study outcome, PD (i.e. within 1 mm). Calibrated examiners collected periodontal probing measurements for each patient at baseline using a UNC-15 periodontal probe (Hu-Friedy; Chicago, IL, USA) for all teeth except third molars and at six sites per tooth. Probing measurements included PD, CAL, BOP, plaque index (PI)^21^ and GI.^13^ When a PD or CAL measurement fell between two millimeter readings, the clinical examiner rounded down and recorded the lower of the two readings. In addition, a multi-pass strategy was employed such that all sites with PD measuring ≥5 mm for any time point had two PD readings recorded (multiple-pass probing measurements).^26^ If the two PD measures were within 1 mm, they were averaged. If the two PD measures were outside of 1 mm, a third PD measure was recorded, and the closer two of the three PD measures were averaged. In this manner, the clinical examiner identified two to four ‘treatment sites’ for each patient with mean PD ≥6 mm and BOP occurring at separate teeth.

### Patient Treatments, Study Groups, Randomization, and Allocation Concealment

All patients received full-mouth SRP using a combination of hand curettes and ultrasonic instruments with local anesthesia. SRP and study group allocation (randomization) were performed by treating clinicians who were separate from the study’s calibrated examiners for concealment or blinding purposes. SRP was completed within one or two sessions during the two weeks following the baseline exam. Treating clinicians then randomized patients to either adjunctive botanical patch devices or no additional treatment (control, SRP alone). Patients allocated to the SRP plus botanical patches (unblinded) were instructed on device applications to the two to four identified treatment sites via demonstration with a mirror and pictorial oral ‘map’. Patients were instructed to not brush or floss while the botanical patches were in place, or to eat for one hour after patch applications. Patients re-applied botanical patch devices to the identified treatment sites for two additional times on day 0 (6 h apart) and then once per day on days 1-6; hence, patient treatment with the botanical patch devices spanned one week following completion of SRP. Study devices were spontaneously shed or removed by patients between 2 and 2.5 h after each application.

Patients were recalled to the study center at 1, 2, and 3 months. The same calibrated, blinded examiners remeasured PD, CAL, BOP, PI and GI. Adverse events were monitored and categorized by system. Botanical patch use and compliance were evaluated via collection of unused study devices.

### Sample Size, Randomization and Statistical Plan

For the statistical plan, the patient was the unit of measure. Patient demographic factors (i.e. gender, age, smoking status, history of diabetes or cardiovascular disease, and number of treatment sites) were summarized as number, percent per group or mean (standard deviation [SD], or standard error [SE]). Periodontal probing measures were averaged across the identified treatment sites and patients using the intent-to-treat principle. In general, intergroup differences in categorical outcomes were evaluated using the Chi^2^ test. Differences in the primary and secondary probing parameters were assessed using ANOVA for baseline parameters and analysis of covariance (ANCOVA controlling for baseline measures) for parameters at 1, 2 and 3 months. Secondary analyses were conducted on smoking vs nonsmoking subpopulations and patients with severe periodontitis (baseline PD ≥7 mm for treatment sites). Odds ratios for pocket resolution (PD <5.0 mm) at 1, 2 or 3 months were calculated using a logistic regression analysis. Adverse events were stratified by body system and group. All analyses were conducted using SAS 9.4 software (SAS Institute; Cary, NC, USA).

## Results

### Patient Enrollment, Demographics and Adverse Events

Of the 80 patients enrolled, two were withdrawn from the trial (one due to non-compliance with study visits and another for antibiotic and steroid medication use). Four patients did not complete the trial (one due to a non-study related injury and three lost to follow-up).

Baseline patient demographics are listed in Table 1. Mean patient ages for the botanical patch group and the SRP control group were 47.3 and 48.0 years, respectively. Whereas 55% of botanical patch patients were female, 45% of control patients were female. This minor gender difference between the groups was not statistically significant. Fifty percent of both groups were African American. Both groups exhibited similar mean body mass indices (approximately 28 kg/m^2^) and comparable numbers with histories of diabetes or cardiovascular disease. Patients in both groups presented with a mean of 3.2 treatment sites at baseline measuring ≥6 mm in PD and with BOP. Treatment groups were balanced with respect to baseline periodontal status with the two groups (Table 1). Mean baseline whole-mouth PD was 3.17 mm for the control group and 2.99 mm for the botanical patch group. In addition, both groups exhibited similar extent scores for PD ≥4 mm and extent scores for CAL ≥3 mm. Chi^2^ testing indicated no statistically significant differences in whole-mouth measures of baseline periodontal status between the two groups. In contrast, groups were not balanced for smoking status. Notably, 12 patients in the control group (30%) were classified as smokers vs five patients (12.5%) in the botanical patch group. This difference was statistically significant (Chi^2^ test; p = 0.002). The clustering of smokers within the control group further prompted the investigator team to stratify for smoking status in the analysis plan for primary and secondary clinical endpoints.

**Table 1:** Subject baseline demographics by treatment group

Demographic factor	SRP alone	Botanical patch	p-value
Mean (SD) Age (years)	48.0 (12.3)	47.3 (12.1)	0.78
Female Male	21 (44.7%) 19 (57.6%)	26 (55.3%) 14 (42.4%)	0.26
African American Caucasian Other	19 (50%) 20 (54.1%) 1 (20.0%)	19 (50%) 17 (46.0%) 4 (80.0%)	0.36
Smoker Nonsmoker	12 (70.6%) 28 (44.4%)	5 (29.4%) 35 (56.6%)	0.002
Diabetes history No diabetes history	2 (50.0%) 38 (50.0%)	2 (50.0%) 38 (50.0%)	1.00
Cardiovascular history No dardiovascular history	8 (38.1%) 32 (54.2%)	13 (61.9%) 27 (45.8%)	0.20
Mean (SD) BMI (kg/m^2^)	28.0 (6.2)	28.9 (0.9)	0.52
Mean (SD) number of treatment sites	3.2 (0.9)	3.2 (0.9)	0.80
Whole mouth mean (SE) PD (mm)	3.17 (0.09)	2.99 (0.09)	0.15
Whole-mouth mean (SE) extent PD ≥4 mm (%)	28.5 (2.47)	24.8 (2.47)	0.30
Whole-mouth mean (SE) extent CAL ≥3 mm (%)	50.1 (4.57)	41.5 (4.57)	0.19

There were 28 adverse events reported in the trial. None was serious. Sixteen of the adverse events were due to oral changes (i.e. worsening of periodontal status or PD deepening ≥3 mm from baseline). These 16 adverse events were limited to eight patients (five in the adjunctive botanical patch group and three in the SRP alone group). Only one of these oral adverse events was localized to an identified treatment site. The remaining 12 adverse events were scattered among the body systems, and were not statistically significantly different in incidence between the two groups.

### Clinical Periodontal Outcomes

Table 2 summarizes mean (SE) periodontal probing parameters derived from the identified treatment sites for all patients and all study visits. Both treatment groups overall exhibited improvements in probing parameters (from baseline) with study treatments. These improvements persisted over the 3-month period within the two groups.

**Table 2:** Mean (SE) periodontal probing parameters (treatment sites) for all subjects and visits, stratified by treatment group

Pocket depth (mm)	Baseline	1 month	2 months	3 months
			
SRP alone	6.58 (0.11)	5.74 (0.09)	5.61 (0.10)	5.23 (0.11)
Botanical patch	6.36 (0.11)	5.45 (0.09)	5.27 (0.10)	5.10 (0.11)
p-value	0.16	0.02	0.02	0.43
Clinical attachment level (mm)				
SRP alone	5.91 (0.20)	5.21 (0.11)	5.13 (0.11)	4.49 (0.13)
Botanical patch	5.23 (0.20)	4.81 (0.11)	4.73 (0.12)	4.47 (0.14)
p-value	0.02	0.01	0.02	0.94
BOP (%)				
SRP alone	95.9 (1.68)	86.0 (3.06)	76.4 (3.43)	74.8 (3.63)
Botanical patch	96.8 (1.65)	87.2 (3.10)	88.2 (3.47)	85.6 (3.67)
p-value	0.69	0.80	0.02	0.02
Plaque index				
SRP alone	1.39 (0.06)	1.01 (0.05)	0.92 (0.05)	0.98 (0.06)
Botanical patch	1.33 (0.06)	0.99 (0.05)	1.03 (0.05)	0.99 (0.06)
p-value	0.48	0.81	0.15	0.91
Gingival index				
SRP alone	1.32 (0.04)	1.09 (0.03)	1.05 (0.04)	1.05 (0.03)
Botanical patch	1.25 (0.04)	1.16 (0.03)	1.15 (0.04)	1.01 (0.03)
p-value	0.25	0.10	0.08	0.38

ANOVA for baseline and ANCOVA for visits at 1, 2 and 3 months.

For the primary outcome variable, patients treated with adjunctive botanical patch devices exhibited significantly reduced PD at 1 and 2 months (p < 0.05) vs SRP alone. This clinical trend continued into the third month but did not reach statistical significance (p = 0.08). The magnitude of the mean difference in PD was approximately 0.30 mm between the groups for 1 and 2 months.

For the secondary outcome, patients allocated to the control group showed significantly greater mean values for CAL at baseline (5.91 mm) as compared to the botanical patch group (5.23 mm, p < 0.05). When an ANCOVA was performed controlling for baseline levels, significantly improved CAL means were detected for the botanical patch group at 1 and 2 months (4.81 mm and 4.73 mm, respectively) vs the control patients (5.21 mm and 5.13 mm, respectively). Mean CAL improved in the botanical patch group at 3 months and relapsed for the control group; however, no significant intergroup differences in CAL were detected at 3 months.

Although both groups showed overall improvements in mean BOP, PI, and GI at 1–3 months, ANCOVA testing indicated no statistically significant intergroup differences for these tertiary outcomes, with the exception of BOP scores. Although percent bleeding scores were statistically significantly lower in the control group at 2 and 3 months (p < 0.05), these mean percent scores were based only on the two to four treatment sites per patient and were high for both groups (≥75%).

Table 3 lists mean probing parameters derived from treatment sites for nonsmoking patients in the two groups. Regarding the PD and CAL changes among nonsmokers, both treatment groups showed continued improvements in PD and CAL from 1–3 months; however, statistically significant intergroup differences in PD and CAL in favor of adjunctive botanical patch therapy were greater (range, 0.40–0.50 mm) among this nonsmoking sub-cohort (p < 0.05 for PD at 1 month, and p < 0.01 for PD at 2 months and CAL at 1 and 2 months, respectively). The mean PD at 3 months for nonsmokers was 4.93 mm with botanical patch treatment vs 5.22 mm for SRP alone. The intergroup difference at 3 months for nonsmokers was borderline statistically significant (p = 0.08), and likely affected by the limited sample size. No statistically significant mean differences for BOP, PI or GI were detected between the nonsmoking groups for any of the time points with the exception of a statistically significantly increased BOP score for the botanical-patch nonsmoking patients at 3 months.

**Table 3:** Mean (SE) periodontal probing parameters (treatment sites) for nonsmoking subjects, stratified by treatment group

Pocket depth (mm)	Baseline	1 month	2 months	3 months
			
SRP alone	6.28 (0.12)	5.60 (0.10)	5.49 (0.11)	5.22 (0.12)
Botanical patch	6.36 (0.11)	5.29 (0.10)	5.05 (0.10)	4.93 (0.11)
p-value	0.58	0.03	0.004	0.08
Clinical attachment level (mm)				
SRP alone	5.24 (0.20)	4.87 (0.12)	4.79 (0.13)	4.39 (0.14)
Botanical patch	5.13 (0.18)	4.37 (0.12)	4.25 (0.12)	4.15 (0.13)
p-value	0.68	0.004	0.002	0.21
BOP (%)				
SRP alone	98.9 (1.31)	88.4 (3.37)	82.9 (3.72)	75.8 (4.07)
Botanical patch	98.2 (1.14)	89.5 (3.08)	88.9 (3.27)	88.8 (3.59)
p-value	0.73	0.81	0.23	0.02
Plaque index				
SRP alone	1.28 (0.07)	0.97 (0.06)	0.86 (0.06)	0.84 (0.07)
Botanical patch	1.29 (0.06)	0.96 (0.05)	1.01 (0.06)	0.95 (0.06)
p-value	0.91	0.89	0.07	0.21
Gingival index				
SRP alone	1.29 (0.05)	1.08 (0.04)	1.05 (0.05)	1.01 (0.04)
Botanical patch	1.24 (0.05)	1.15 (0.04)	1.17 (0.05)	1.01 (0.03)
p-value	0.50	0.19	0.07	1.00

ANOVA for baseline and ANCOVA for visits at 1, 2 and 3 months.

Table 4 summarizes mean periodontal probing parameters for the patients identified as smokers (n = 17). While smoking patients allocated to the control arm exhibited statistically significantly greater mean PD at baseline (7.34 mm, p < 0.05) compared to patients allocated to botanical patches (6.34 mm), an ANCOVA controlling for baseline PD revealed no statistically significant intergroup difference at 1, 2 or 3 months for PD or any of the other measured periodontal parameters, with the exception of GI at 2 months. Here, smokers treated with the botanical patch exhibited a statistically significantly lower mean GI score (0.73) vs smokers treated with SRP alone (mean GI of 1.05, p = 0.03) at 2 months.

**Table 4:** Mean (SE) periodontal probing parameters (treatment sites) for subjects who smoke, stratified by treatment group

Pocket depth (mm)	Baseline	1 month	2 months	3 months
			
SRP alone	7.34 (0.22)	6.32 (0.19)	6.27 (0.19)	5.47 (0.27)
Botanical patch	6.36 (0.34)	5.97 (0.29)	6.60 (0.42)	6.49 (0.61)
p-value	0.02	0.34	0.49	0.14
Clinical attachment level (mm)				
SRP alone	7.57 (0.44)	6.61 (0.23)	6.91 (0.24)	5.89 (0.31)
Botanical patch	6.00 (0.70)	6.62 (0.36)	7.28 (0.52)	6.81 (0.68)
p-value	0.06	0.98	0.53	0.23
BOP (%)				
SRP alone	88.9 (5.52)	79.0 (7.20)	59.7 (8.23)	67.4 (7.93)
Botanical patch	85.7 (8.86)	72.0 (11.2)	86.3 (18.2)	43.9 (17.5)
p-value	0.76	0.59	0.17	0.23
Plaque index				
SRP alone	1.64 (0.11)	1.14 (0.09)	1.12 (0.09)	1.36 (0.10)
Botanical patch	1.64 (0.19)	1.14 (0.15)	0.94 (0.21)	1.25 (0.24)
p-value	0.99	1.00	0.45	0.69
Gingival index				
SRP alone	1.39 (0.09)	1.10 (0.06)	1.05 (0.06)	1.14 (0.06)
Botanical patch	1.31 (0.15)	1.26 (0.10)	0.73 (0.14)	0.87 (0.13)
p-value	0.61	0.21	0.03	0.08

ANOVA for baseline and ANCOVA for visits at 1, 2 and 3 months.

Similarly, Table 5 lists changes in patient means derived from the deepest sites measuring ≥7 mm in PD at baseline. While patients treated with adjunctive botanical patches exhibited a mean PD reduction of 0.99 mm at 1 month, control patients exhibited a PD reduction of 0.39 mm (borderline significant, p = 0.07). ANOVA and ANCOVA testing indicated no significant intergroup differences for any of the probing parameters or time points for the deepest site-patient means.

**Table 5:** Mean (SE) periodontal probing parameters for sites with severe pocketing (baseline PD ≥7 mm) stratified by treatment group

Pocket depth (mm)	Baseline	1 month	2 months	3 months
			
SRP alone	7.87 (0.19)	7.48 (0.16)	7.10 (0.20)	6.71 (0.25)
Botanical patch	8.00 (0.24)	7.01 (0.20)	7.08 (0.25)	6.50 (0.31)
p-value	0.69	0.07	0.97	0.58
Clinical attachment level (mm)				
SRP alone	7.85 (0.38)	7.48 (0.20)	7.30 (0.23)	6.80 (0.28)
Botanical patch	7.21 (0.46)	6.94 (0.26)	7.13 (0.29)	6.72 (0.28)
p-value	0.28	0.11	0.65	0.85
BOP (%)				
SRP alone	92.1 (3.40)	82.1 (5.81)	71.6 (6.56)	82.7 (4.83)
Botanical patch	100.0 (4.03)	90.9 (7.34)	89.7 (8.18)	97.4 (6.01)
p-value	0.14	0.35	0.09	0.06
Plaque index				
SRP alone	1.37 (0.11)	0.99 (0.09)	0.95 (0.08)	1.06 (0.11)
Botanical patch	1.43 (0.14)	0.98 (0.11)	0.95 (0.10)	1.07 (0.13)
p-value	0.72	0.92	0.99	0.92
Gingival index				
SRP alone	1.37 (0.08)	1.10 (0.06)	1.10 (0.06)	1.12 (0.05)
Botanical patch	1.30 (0.10)	1.17 (0.08)	1.13 (0.07)	0.96 (0.07)
p-value	0.60	0.45	0.78	0.07

ANOVA for baseline and ANCOVA for visits at 1, 2 and 3 months.

When logistical regression analyses were performed for pocket resolution (PD <5 mm at 1, 2 or 3 months) for all patients, nonsmokers and smokers, odds ratios ranged between 0.53 and 1.70 (data not shown). The confidence intervals for all of the odds ratios included 1.00; hence, no statistically significant differences in the odds for pocket resolution were detected when using this model.

## Discussion

The data from this randomized, controlled clinical trial document the clinical benefits of botanical patch devices used in combination with SRP for the treatment of periodontitis, especially for patients who do not smoke or use tobacco products. Statistically significant improvements in PD and CAL were consistently observed at 1 and 2 months in the trial following adjunctive treatment with the botanical patches as compared to SRP alone. When controlling for mean baseline PD or CAL, the statistically significant improvements in probing parameters with the botanical patch devices were detected for the overall trial population and for nonsmokers at 1 and 2 months. Patients randomized to botanical patches were instructed to administer the study devices to designated treatment sites three times on day 0 (following the last SRP session) and then once daily for remainder of the week (days 1–6). The data from this clinical trial indicate that patients can be compliant with instructions and can apply the patch devices in a site-specific manner as prescribed for one week, with statistically significant clinical effects measured over 2 months. The absence of intergroup differences in probing parameters by 3 months coincides with a usual maintenance interval for a patient with periodontitis.

There are several limitations to this clinical trial, including the moderate sample size (n = 40 patients per group), short trial duration, absence of stratification strategies at the time of randomization, and the clustering of smokers among the control group. Patients randomized for botanical patches were comparable to control patients with regard to baseline mean and extent PD scores but not baseline mean CAL. Although the data indicate short-term clinical improvements in probing parameters with adjunctive botanical patch use, these findings may not be generalizable to broader populations because of the limited sample size. Also, while the patch devices contain botanical extracts with anti-inflammatory properties, the data from this clinical trial did not reveal consistent or significant reductions in outcomes traditionally related to inflammation such as BOP or GI for the adjunctive patch use vs controls.

Other published trials have consistently demonstrated the efficacy of adjunctive therapies for managing periodontitis. For example, Williams et al^26^ reported that SRP plus minocycline microspheres significantly improved PD at 1, 3, 6 and 9 months in periodontitis patients as compared to SRP alone or SRP plus placebo microspheres. CAL changes were not reported for this pivotal study. In contrast, Jeffcoat et al^10^ showed that adjunctive treatment with chlorhexidine chips resulted in significant PD reductions at only 6 weeks but significant CAL gains at 3 and 6 months as compared to SRP alone. In addition, an 8.5% doxycycline hyclate gel monotherapy (i.e. in the absence of SRP) produced equivalent PD reductions over 9 months and significantly greater CAL gains at 6–9 as compared to SRP controls.^7^ Lastly, periodontitis patients taking sub-antimicrobial dose doxycycline (20 mg) twice daily after SRP exhibited statistically significantly greater PD reductions and CAL gains at 3, 6 and 9 months (i.e. for sites with baseline PD = 4–6 mm) as compared to patients taking a placebo.^2^ These phase-III clinical trials featured large sample sizes (range, 190–748 patients), spanned 9 months, and followed design specifications in support of new drug applications (NDAs) with the US FDA. Observed 3-month PD reductions within these trials ranged from 0.8 mm for chlorhexidine-gelatin chips to 1.3 mm for minocycline microspheres. Three-month CAL gains (when reported) ranged from 0.6 mm with chlorhexidine-gelatin chip therapy to 1.0 mm with low dose doxycycline therapy. Limitations of these adjunctive drug approaches include concerns over antibiotic resistance development for the minocycline microspheres and doxycycline gel, and the necessity for chronic peroral dosing with sub-antimicrobial dose doxycycline.

In contrast, the topical botanical patch evaluated in this clinical trial constitutes an FDA and EU-approved device (not a drug) for intraoral use and whose components are all generally recognized as safe (GRAS). The PD reductions and CAL gains with the botanical patch observed at 3 months were 1.3 and 0.8 mm respectively, and were comparable to the improvements reported for minocycline microspheres, chlorhexidine chips, doxycycline hyclate gel and sub-antimicrobial dose doxycycline as detailed above. Uniquely, the botanical patches produced significant improvements in both PD and CAL as soon as 1-2 months post-SRP, according to the one-week application protocol. In addition, the botanical patch devices do not present inherent problems such as antibiotic resistance development or chronic dosing posed by the approved drug adjuncts for managing chronic periodontitis.

An animal study conducted by Chaushu et al^3^ indicated that botanical patch devices may enhance tissue repair and wound healing. Accordingly, the investigators created surgical wounds in the edentulous maxillae of laboratory rats and histologically evaluated wound healing in four randomized groups (i.e. surgery plus botanical patch devices, surgery plus placebo patch, surgery but no patch, and no surgery and no patch). Botanical patch devices vs placebo patches were applied to the oral wounds twice daily for three days according to the randomization scheme. Results showed that surgical wounds treated with botanical patches resulted in significantly increased wound closure (epithelialization), collagen deposition, and angiogenesis over 12 days vs wounds treated with placebo devices or no patches. Hence, the improvements in PD and CAL observed in the present human trial are most likely related to enhanced wound healing events at the cellular level, secondary to the botanical patch applications.

In 2015, the American Dental Association Council of Scientific Affairs convened a scientific panel to conduct a systematic review and meta-analysis on nonsurgical treatment of periodontitis including SRP with and without adjuncts.^22^ CAL was selected as the primary outcome for the review and meta-analysis. The panel identified 72 citations (i.e. published prior to July 2014) on the effectiveness of SRP, systemic antimicrobials, locally administered antimicrobials (minocycline microspheres, chlorhexidine chips and doxycycline hyclate gel), systemic host modulator (sub-antimicrobial dose doxycycline), and a variety of nonsurgical lasers (photodynamic therapy with a diode laser, a diode laser, neodymium:yttrium-aluminum-garnet lasers, and erbium lasers). With a moderate level of certainty, the panel found that SRP alone produced mean CAL gains of approximately 0.5 mm in patients. Combinations of SRP with assorted adjuncts resulted in mean CAL improvements ranging between 0.2–0.6 mm over SRP alone. The panel determined that systemic antimicrobials, sub-antimicrobial dose doxycycline, chlorhexidine chips, and photodynamic therapy with a diode laser produced adjunctive benefits beyond SRP with a moderate level of certainty. Given the limitations of the available evidence, the panel determined that there was a low level of certainty regarding the benefits of the other reviewed adjunctive therapies.

The present clinical trial of botanical patch use in patients with periodontitis adds to this body of evidence on adjunctive therapies. The findings reiterate the importance of SRP, removal of the etiologic bacteria, and disruption of the biofilm in the initial phase of treatment. For the overall trial population and the subgroup of nonsmokers, the combination of SRP plus topical botanical patch application resulted in statistically significant PD reductions and CAL gains at 1 and 2 months as compared to SRP alone.

## Conclusion

The data from this clinical trial indicate short-term improvements in probing parameters with the botanical patch device when used adjunctively with SRP, especially in non-smoking periodontitis patients.
